# Catalase Predicts In-Hospital Mortality after Out-of-Hospital Cardiac Arrest

**DOI:** 10.3390/jcm10173906

**Published:** 2021-08-30

**Authors:** Anton Früh, Andrea Bileck, Besnik Muqaku, Raphael Wurm, Benjamin Neuditschko, Henrike Arfsten, Lukas Galli, Lukas Kriechbaumer, Pia Hubner, Georg Goliasch, Gottfried Heinz, Michael Holzer, Fritz Sterz, Christopher Adlbrecht, Christopher Gerner, Klaus Distelmaier

**Affiliations:** 1 Department of Internal Medicine II, Medical University of Vienna, 1090 Vienna, Austria; antonfrueh@aol.com (A.F.); henrike.arfsten@meduniwien.ac.at (H.A.); lukas.galli@meduniwien.ac.at (L.G.); georg.golisch@meduniwien.ac.at (G.G.); gottfried.heinz@meduniwien.ac.at (G.H.); klaus.distelmaier@meduniwien.ac.at (K.D.); 2 Department of Analytical Chemistry, Faculty of Chemistry, University of Vienna, 1090 Vienna, Austria; andrea.bileck@univie.ac.at (A.B.); besnik.muqaku@univie.ac.at (B.M.); benjamin.neuditschko@univie.ac.at (B.N.); 3 Joint Metabolome Facility, Faculty of Chemistry, University of Vienna, 1090 Vienna, Austria; 4 Department of Neurology, Medical University of Vienna, 1090 Vienna, Austria; raphael.wurm@meduniwien.ac.at; 5 Department of Inorganic Chemistry, Faculty of Chemistry, University of Vienna, 1090 Vienna, Austria; 6 University Clinic of Orthopedics, Paracelsus Medical University Salzburg, 5020 Salzburg, Austria; lukikriechbaumer@hotmail.com; 7 Department of Emergency Medicine, Medical University of Vienna, 1090 Vienna, Austria; pia.hubner@meduniwien.ac.at (P.H.); michael.holzer@meduniwien.ac.at (M.H.); fritz.sterz@meduniwien.ac.at (F.S.); 8 Imed19-Privat, Private Clinical Research Center, 1190 Vienna, Austria; c.adlbrecht@imed19.at

**Keywords:** catalase, MRM, out-of-hospital cardiac arrest, survival, targeted proteomics

## Abstract

The generation of harmful reactive oxygen species (ROS), including hydrogen peroxide, in out-of-hospital cardiac arrest (OHCA) survivors causes systemic ischemia/reperfusion injury that may lead to multiple organ dysfunction and mortality. We hypothesized that the antioxidant enzyme catalase may attenuate these pathophysiological processes after cardiac arrest. Therefore, we aimed to analyze the predictive value of catalase levels for mortality in OHCA survivors. In a prospective, single-center study, catalase levels were determined in OHCA survivors 48 h after the return of spontaneous circulation. Thirty-day mortality was defined as the study end point. A total of 96 OHCA survivors were enrolled, of whom 26% (*n* = 25) died within the first 30 days after OHCA. The median plasma intensity levels (log_2_) of catalase were 8.25 (IQR 7.64–8.81). Plasma levels of catalase were found to be associated with mortality, with an adjusted HR of 2.13 (95% CI 1.07–4.23, *p* = 0.032). A Kaplan–Meier analysis showed a significant increase in 30-day mortality in patients with high catalase plasma levels compared to patients with low catalase levels (*p* = 0.012). High plasma levels of catalase are a strong and independent predictor for 30-day mortality in OHCA survivors. This indicates that ROS-dependent tissue damage is playing a crucial role in fatal outcomes of post-cardiac syndrome patients.

## 1. Introduction

Out-of-hospital cardiac arrest (OHCA) is a major public health concern, affecting annually approximately 300,000 patients in Europe [1]. It is associated with substantial morbidity and mortality rates of up to 90% [2]. Even only about 30% of OHCA patients that reach hospitals with ongoing cardiopulmonary resuscitation (CPR) or return of spontaneous circulation (ROSC) survive for at least 30 days or till hospital discharge [2]. Accurate prediction of neurological outcome and mortality of this patient collective is still difficult and can be enhanced by considering new clinical [3] and laboratory parameters [4].

Patients regaining spontaneous circulation after OHCA suffer from a combination of pathophysiological processes, including brain injury, myocardial dysfunction, systemic ischemia, and reperfusion, and the initial pathology that caused the cardiac arrest. The combination of these ongoing deleterious processes critically affects patient outcome, and is coined as post-cardiac arrest syndrome [5,6]. An optimized risk evaluation in OHCA patients that initially survived the cardiac arrest and are facing the life-threatening post-arrest period may have important implications, both for evaluating further therapeutic strategies and clinical judgment of prognosis.

After OHCA and following ROSC, patients encompass a systemic ischemic and reperfusion response [5,6]. Thereby, hypoxia and reoxygenation processes promote the generation of harmful reactive oxygen species (ROS) as hydrogen peroxide [5,6,7]. Catalase is an antioxidant enzyme that serves to protect cells from toxic effects by catalyzing the defusing of hydrogen peroxide to water and oxygen. This protein occurs in almost all aerobically respiring organisms and is expressed in all human tissues [8]. Although catalase has shown protective effects for brain tissues in cerebral ischemia rat models [9] and overexpression of this enzyme in heart tissues can suppress the ischemic–reperfusion injury of hearts in mice models [10], the role of this enzyme in OHCA survivors has not been investigated yet. We hypothesized that catalase may play a role in pathophysiological processes of patients suffering from post-cardiac arrest syndrome by moderating ROS-dependent tissue damage. Thus, we aimed, in the present study, to determine the impact of catalase levels on survival in OHCA survivors.

## 2. Materials and Methods

This is a sub-study of a prospectively performed single-center study that included OHCA survivors at the Medical University of Vienna between October 2013 and May 2016 [11]. We enrolled unconscious patients after OHCA with a Glasgow Coma Scale of 3 (eye opening 1, verbal response 1, motor response 1) [12] on arrival at the emergency department, and observed them till discharge from the intensive care unit (ICU), as previously described [11]. Inclusion presupposed nontraumatic, normothermic cardiac arrest caused by cardiac disorders, respiratory failures, or hemodynamic or metabolic factors. Patients with previous cardiac arrest, hydrocephalus and shunt artifact, intracerebral hemorrhage, known or coexisting neurological and psychiatric disorders, neoplasms of the central nervous systems, and psychotropic medication were excluded. The study was approved by the Ethics Committee of the Medical University of Vienna (EK 1740/2013) and registered with ClinicalTrials.gov number NCT01960699. All procedures of this study were performed in accordance with the Declaration of Helsinki. In case of awakening, written informed consent was retroactively obtained.

All OHCA patients were treated during cardiopulmonary resuscitation (CPR) and in post-resuscitation management according to current guidelines [13]. Cardiac arrest data sets were recorded according to “Utstein” criteria [14]. All patients were routinely treated with targeted temperature management at 33 °C to 36 °C for 24 h. Venous blood samples of all patients were drawn from peripheral and central vein catheters 48 h after hospital admission in EDTA tubes. The specimens were immediately placed on ice and centrifuged to obtain platelet-poor plasma. Additional plasma samples were stored at −80 °C and were applied for a targeted proteomic approach after thawing. Plasma was gained via centrifugation, depleted using Pierce™ Top 12 Abundant Protein Depletion Spin Columns, and catalase was measured via targeted proteomics, as described [11]. In brief, a multiple reaction monitoring (MRM) assay was established on an Agilent 6490 triple quadrupole mass spectrometer coupled with a nano-chip-LC Agilent Infinity Series HPLC1260 system. Details of the established MRM method to determine protein abundance levels of catalase are summarized in Table 1. Specimens were measured in technical duplicates. The abundance of catalase was calculated as the mean value over these two technical duplicates of all transition signals generated from all peptides belonging to a protein.

Discrete data were described by absolute and relative frequencies and compared between groups using chi-square tests. Continuous data were presented as medians (with interquartile ranges) and compared between groups using the Mann–Whitney U-tests. Quantification and statistical analyses of the mass spectrometry data set were based on label-free quantification (LFQ) values using Perseus software, as previous described [15]. The study population was subdivided into patients with high and low catalase levels. The median of catalase plasma levels has been chosen as the cut-off point. Cox proportional hazard regression analysis was applied to assess the effect of catalase levels on 30-day survival. Results are expressed as hazard ratios (HR) with respective 95% confidence intervals (CI). To account for potential confounding effects, we calculated the risk of death adjusted for age, presence of a shockable rhythm, and time to ROSC. Kaplan–Meier analysis was applied to evaluate the effect of catalase levels on survival and compared using a log-rank test. Two-sided *p*-values < 0.05 were taken to indicate statistical significance. SPSS version 25.0 (IBM Corp, Armonk, NY, USA) and RStudio version 1.4.1717 were used to evaluate the ability to predict the mortality of OHCA patients.

## 3. Results

### 3.1. Baseline Characteristics

We enrolled a total of 100 OHCA survivors. Four patients were excluded because of poor specimen quality. Thus, the final study population consisted of 96 patients, with a median age of 58 years (IQR: 48–69), of whom 25 patients (26%) died within 30 days after OHCA. In 86.5% of cases, the cause of OHCA was of cardiac origin, in 11.5%, of respiratory reasons, and, in 2%, the underlying cause was not identified. In 73% of patients, the first monitored rhythm was shockable. The median time from OHCA to ROSC was 28.00 min (IQR 15.00–44.00). The median plasma intensity levels (log_2_) of catalase were 8.25 (IQR 7.64–8.81). According to the median catalase level, the study population was stratified into patients with high (log_2_ intensity ≥ 8.25) and low catalase levels (log_2_ intensity < 8.25). Detailed baseline characteristics, and characteristics for patients with high and low catalase levels are displayed in Table 2. Characteristics of OHCA survivors and non-survivors are provided in Table S1.

### 3.2. Catalase and 30-Day Mortality of OHCA Survivors

**Figure 1:**
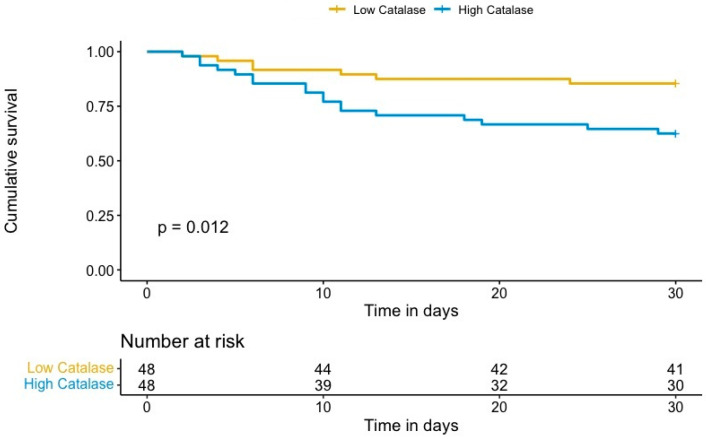
Kaplan–Meier estimates of 30-day mortality for OHCA survivors with high (log_2_ intensity ≥ 8.25) and low catalase levels (log_2_ intensity < 8.25).

We identified a significant association between catalase plasma levels and 30-day mortality in OHCA survivors, with an unadjusted hazard ratio (HR) per one standard deviation (SD) of 2.26 (95% confidence interval (CI) 1.49–3.42; *p* < 0.001). To account for potential confounding effects, we adjusted the risk of 30-day mortality for covariates previously associated with outcome in OHCA, including age, presence of a shockable rhythm, and time to ROSC [16]. The results persisted after multivariate adjustment, with an adjusted HR per one SD of 2.13 (95% CI 1.07–4.23, *p* = 0.032). Kaplan–Meier analysis revealed a significant increase in 30-day mortality in patients with high catalase plasma levels compared to patients with low catalase levels (*p* = 0.012, log rank, Figure 1). Univariate and multivariable analyses of known clinical risk factors of mortality after OHCA are summarized in Table 3.

## 4. Discussion

This study identified plasma catalase levels as a strong and independent predictor of poor mortality in OHCA patients. Short-term survival was significantly reduced in patients with high catalase levels, determined 48 h after the event. Imbalance between generation of ROS and antioxidant body defense results in oxidative stress that affects the clinical outcome and can be used for the prediction of mortality of critically ill patients [17]. Enhanced levels of oxidative stress can be measured during various critical illness situations, such as cardiovascular disorders, cardiogenic shock, organ dysfunctions, and after cardiac arrest [5,18,19]. Thereby, the function of antioxidant counter-reactions, e.g., via HDL, is associated with the survival of this patient population [17]. One of the harmful ROS metabolites that is generated during post-cardiac arrest syndrome is hydrogen peroxide [5]. As catalase defuses hydrogen peroxide to harmless water and oxygen, we hypothesized that this enzyme may play a role in the pathophysiological processes of OHCA survivors. Furthermore, we suggested that this antioxidant body defense protein may be upregulated to counter oxidative stress and may, therefore, be a predictor for poor outcome.

After ischemic events followed by reperfusion, a burst of hydrogen peroxide can be measured in vitro within the first five minutes [20]. However, there is no experimental and clinical evidence demonstrating the kinetic response of ROS-burden-induced catalase upregulation. Because 12 to 72 h after cardiac arrest injury, pathways are still active and aggressive treatment on OHCA patients is typically instituted [5], we determined catalase 48 h after OHCA.

OHCA is a leading cause of death in industrial countries [21]. After successful resuscitation, patients suffer from post-cardiac syndrome, resulting in high mortality rates [2,6]. Better prediction of survival in these patients may have significant implications, both for evaluating further therapeutic strategies and clinical judgment of prognosis. Knowledge about prognosis may beneficially guide the information of family members, which plays an important role for clinicians at the ICU [22]. Furthermore, a reliable prognosis can help to avoid unpromising therapeutic actions that may even impair the quality of the remaining life for the patient, the family members, and the caregiver [23].

In this study, we measured high levels of catalase in the serum samples of OHCA survivors with a poor short-time mortality outcome. This could point to a pronounced hypoxia and ROS-mediated tissue injury burden of these patients. Catalase seems to be increased to dismantle oxidative stress. Therefore, higher levels of this protein may implicate stronger occurrence of hypoxic and related reperfusion events. Furthermore, the data implicate an important role of catalase in pathophysiological processes of critically ill patients. This could lead to a better understanding of fatal processes in this patient population and, therefore, may be helpful to identify novel therapeutic strategies.

Targeted temperature management represents an established therapeutic strategy to improve outcome in OHCA survivors [24]. The underlying mechanisms are not fully understood, but one of its effects is to reduce oxidative damage and alter antioxidant defense mechanisms [25]. Additionally, the administration of antioxidant agents as adjunctive therapies to attenuate ischemia–reperfusion injury in patients with acute myocardial infarction is suggested to have beneficial effects on clinical outcomes [19]. Therefore, it is tempting to speculate that augmented antioxidant therapy may represent a promising treatment approach in OHCA survivors, in particular in those patients with elevated catalase levels. Thereby, high catalase levels may point to an imbalance between oxidant and antioxidant. Therefore, determination of catalase may allow the identification of patients benefiting from antioxidant therapies, supporting the current trend of personalized medicine [26].

Moreover, anesthetic propofol is a free radical scavenger that has cytoprotective effects during hypoxia [27]. It has been postulated that these cytoprotective effects are inter alia mediated indirectly via enhancement of the activity of catalase [28]. Therefore, augmenting of catalase activity in patients may be helpful in the therapy of patients suffering from post-cardiac arrest syndrome.

Patients with acatalasemia and hypocatalasemia suffering from hereditary insufficient catalase activity are associated with several diseases, such as essential hypertension [29]. It could be interesting to further explore the OHCA mortality of this patient collective compared to non-catalase-deficient patients.

The present study is inherently limited by its design as post hoc analysis of a prospective trial. Larger, prospective studies are needed to verify our results. Further investigations and experiments are required to elucidate the role of catalase during post-cardiac arrest syndrome and to evaluate the effects of antioxidant therapy after cardiac arrest. Dynamic changes of catalase and further laboratory values were not investigated in this study, and should be addressed in additional studies. To further investigate the hypothesized imbalance between oxidant and antioxidant levels in OHCA survivors with high serum catalase, indicating even higher oxidant levels in these patients, measurement of oxidant levels, such as hydrogen peroxide or anion superoxide, may be tempting.

The high incidence of patients suffering from OHCA combined with high mortality rates, underlines the continuing clinical need to estimate mortality outcome predication models and the development of new therapeutic strategies. We, therefore, determined catalase as a strong and independent predictor for poor outcome of 30-day mortality. This indicates that ROS-dependent tissue damage may play a crucial role in fatal outcomes of post-cardiac syndrome patients. Antioxidant therapy via elevation of catalase activity may, therefore, be a basic therapeutic idea to moderate post-cardiac arrest syndrome and may improve survival rates of patients suffering from OHCA.

## Figures and Tables

**Table 1:** Multiple reaction monitoring (MRM) assay. Transitions (Product m/z) for catalase measurement are listed.

UniProt ID	Protein Name	Gene Name	Peptide Sequence	Precursor m/z	Precursor Charge	Product m/z	Product Charge	Fragment Ion	Collision Energy
P04040	Catalase	CAT	FNTANDDNVTQVR	747.35	2	1131.54	1	y10	22.1
P04040	Catalase	CAT	FNTANDDNVTQVR	747.35	2	1060.5	1	y9	22.1
P04040	Catalase	CAT	FNTANDDNVTQVR	747.35	2	831.43	1	y7	22.1

**Table 2:** Patient baseline characteristics of total study population (*n* = 96), and for patients with low (<8.25) and high (≥8.25) catalase abundance values (Log_2_), measured with multiple reaction monitoring analysis.

	Total Study Population (*n* = 96)	Low Catalase (*n* = 48)	High Catalase (*n* = 48)	*p*-Value
Age, yr, median (IQR)	58 (48–69)	56 (46–69)	59 (50–69)	0.548
Female sex, *n* (%)	22 (22.9)	8 (16.7)	14 (29.2)	0.145
Cardiac arrest witnessed, *n* (%)	79 (82.3)	40 (83.3)	39 (81.3)	0.789
**Location of collapse**				0.66
Private, *n* (%)	47 (49.0)	28 (58.3)	19 (39.6)	
Public, *n* (%)	49 (51.0)	20 (41.7)	29 (60.4)	
**Cause of cardiac arrest (%)**				0.629
Cardiac, *n* (%)	83 (86.5)	43 (89.6)	40 (83.3)	
Pulmonary, *n* (%)	11 (11.5)	4 (8.3)	7 (14.6)	
Unknown, *n* (%)	2 (2.1)	1 (2.1)	1 (2.1)	
**First monitored rhythm (%)**				0.234
Shockable rhythm				
Ventricular fibrillation, *n* (%)	69 (71.9)	38 (79.2)	31 (64.6)	
Ventricular tachycardia, *n* (%)	1 (1.0)	1 (2.1)	0 (0.0)	
Asystole, *n* (%)	9 (9.4)	2 (4.2)	7 (14.6)	
Pulseless electrical activity, *n* (%)	13 (13.5)	6 (12.5)	7 (14.6)	
Unknown first rhythm, *n* (%)	4 (4.2)	1 (2.1)	3 (6.3)	
**Time from cardiac arrest to event—in minutes**				
Start of life support, min, median (IQR)	11 (3–14)	11 (1–14)	11 (4–15)	0.926
Return of spontaneous circulation, min, median (IQR)	28 (15–44)	27 (15–43)	32 (15–46)	0.755
Administration of epinephrine, min, median (IQR)	11 (9–15)	12 (11–15)	11 (5–15)	0.263
Dose of epinephrine administered, mg, median (IQR)	3 (2–5)	3 (1–5)	4 (3–5)	0.225
**Mode of cooling**				0.509
Invasive, *n* (%)	71 (74.0)	34 (70.8)	37 (77.1)	
Non-invasive, *n* (%)	13 (13.5)	8 (16.7)	5 (10.4)	
Combined, *n* (%)	8 (8.3)	5 (10.4)	3 (6.3)	
Unknown, *n* (%)	4 (4.2)	1 (2.1)	3 (6.3)	
**Medical history**				
Hypertension, *n* (%)	35 (36.5)	20 (41.7)	15 (31.3)	0.289
History of smoking, *n* (%)	37 (38.5)	18 (37.5)	19 (39.6)	0.834
Diabetes, *n* (%)	15 (15.6)	6 (12.5)	9 (18.8)	0.374
Acute myocardial infarction, *n* (%)	16 (16.7)	10 (20.8)	6 (12.5)	0.273
COPD, *n* (%)	10 (10.4)	3 (6.3)	7 (14.6)	0.181
Coronary artery disease, *n* (%)	18 (18.8)	11 (22.9)	7 (14.6)	0.296
**Laboratory values at admission**				
Catalase, median (IQR)	8.25 (7.64–8.81)			
pO2 mmHg, median (IQR)	61.15 (25.68–215.00)	61.15 (24.80–61.15)	65.5 (27.50–191.00)	0.793
pCO2 mmHg, median (IQR)	15.70 (7.48–23.85)	12.25 (7.33–21.10)	16.10 (10.25–26.00)	0.155
pH, median (IQR)	7.18 (7.08–7.23)	7.18 (7.11–7.25)	7.16 (7.07–7.22)	0.199
Sodium mmol/L, median (IQR)	138 (136–140)	138 (136–140)	138 (136–139)	0.490
Potassium mmol/L, median (IQR)	3.85 (3.48–4.54)	3.73 (3.46–4.70)	3.92 (3.60–4.53)	0.732
Bicarbonate mmol/L, median (IQR)	8.30 (5.88–10.93)	8.50 (4.70–10.93)	8.30 (6.33–11.10)	0.401
Base excess mmol/L, median (IQR)	9.10 (6.00–12.75)	8.40 (4.88–12.55)	9.85 (7.23–12.90)	0.120
Lactate mmol/L, median (IQR)	7.20 (4.90–9.90)	6.70 (4.60–9.55)	7.50 (5.55–10.75)	0.207
Creatinine, mg/dL, median (IQR)	1.27 (1.04–1.54)	1.33 (1.00–1.57)	1.26 (1.05–1.50)	0.573
Blood urea nitrate, mg/dL, median (IQR)	15.10 (12.00–19.90)	15.50 (10.90–20.05)	14.95 (12.38–19.68)	0.994
NT-proBNP, pg/mL, median (IQR)	441 (118–1862)	333 (87–2070)	799 (145–1552)	0.595
S100B, µg/L, median (IQR)	0.10 (0.06–0.21)	0.09 (0.06–0.15)	0.12 (0.08–0.30)	0.025
Neuron specific enolase, µg/L, median (IQR)	30.65 (17.85–74.18)	27.80 (14.33–49.05)	35.45 (19.55–87.38)	0.07

Abbreviations: COPD: chronic obstructive pulmonary disease, pO2: partial pressure of oxygen, pCO2: partial pressure of carbon dioxide, IQR: interquartile range, *n*: number, yr: years.

**Table 3:** Univariate and multivariable models for risk factors associated with mortality after out-of-hospital cardiac arrest.

	Univariate Models		Multivariable Models	
Parameter	HR (95% CI)	*p*-value	HR (95% CI)	*p*-value
Catalase	2.26 (1.49–3.42)	<0.001	2.13 (1.07–4.23)	0.032
**Clinical Confounders**				
Age, years *	1.02 (0.99–1.06)	0.130	1.05 (1.00–1.11)	0.052
Presence of a shockable rhythm	0.18 (0.07–0.406)	<0.001	0.35 (0.11–1.06)	0.063
Time to ROSC, min *	1.00 (0.98–1.02)	0.961	1.00 (0.98–1.01)	0.822

* variable was scaled to 1 standard deviation. Multivariable analysis was adjusted for age, presence of a shockable rhythm, time to ROSC, and significant variables were presented by HR and 95% CI. ROSC: return of spontaneous circulation.

## Data Availability

The data presented in this study are available on request from the corresponding author.

## Supplementary Materials

The following are available online at https://www.mdpi.com/article/10.3390/jcm10173906/s1, Table S1: Patient baseline characteristics of total study population (*n* = 96) and for OHCA survivors (*n* = 71) and OHCA non-survivors (*n* = 25).
