# Supplementary material for: Catalase Predicts In-Hospital Mortality after Out-of-Hospital Cardiac Arrest
Source: J Clin Med. 2021 Aug 30;10(17):3906. doi: 10.3390/jcm10173906 (PMC8432041; doi:10.3390/jcm10173906)
Supplement: Supplementary file 1 [file jcm-10-03906-s001.zip › jcm-1346588-supplementary.pdf]

Table S1. Patient baseline characteristics of total study population (n = 96) and for OHCA survivors (n = 71) and OHCA non-survivors (n = 25)

	**Total study population (n = 96)**	**OHCA survivors (n = 71)**	**OHCA non-survivors (n = 25)**	**p-value**
Age, yr, median (IQR)	58 (48-69)	56 (46-68)	60 (51-71)	0.137
Female sex, n (%)	22 (22.9)	15 (21.1)	7 (28.0)	0.482
Cardiac arrest witnessed, n (%)	79 (82.3)	62 (87.3)	17 (68.0)	**0.030**
**Location of collapse**				0.132
Private, n (%)	47 (49.0)	38 (53.5)	16 (64.0)	
Public, n (%)	49 (51.0)	33 (46.5)	9 (36.0)	
**Cause of cardiac arrest (%)**				**0.013**
Cardiac, n (%)	83 (86.5)	65 (91.5)	18 (72.0)	
Pulmonary, n (%)	11 (11.5)	6 (8.5)	5 (20.0)	
Unknown, n (%)	2 (2.1)	0 (0.0)	2 (8.0)	
**First monitored rhythm** (%)				**< 0.001**
Shockable rhythm				
Ventricular fibrillation, n (%)	69 (71.9)	59 (83.1)	10 (40.0)	
Ventricular tachycardia, n (%)	1 (1.0)	1 (1.4)	0 (0.0)	
Asystole, n (%)	9 (9.4)	1 (1.4)	8 (32.0)	
Pulseless electrical activity, n (%)	13 (13.5)	7 (9.9)	6 (24.0)	
Unknown first rhythm, n (%)	4 (4.2)	3 (4.2)	1 (4.0)	
**Time from cardiac arrest to event – in minutes**				
Start of life support, min, median (IQR)	11 (3-14)	11 (8-15)	12 (6-16)	0.394
Return of spontaenous circulation, min, median (IQR)	28 (15-44)	27 (15-45)	34 (18-43)	0.705

Administration of epinephrine, min, median (IQR)	11 (9-15)	11 (8-15)	13 (8-16)	0.648
Dose of epinephrine administered, mg, median (IQR)	3 (2-5)	3 (2-5)	4 (3-6)	0.287
**Mode of cooling**				0.409
Invasive, n (%)	71 (74.0)	51 (71.8)	20 (80.0)	
Non-invasive, n (%)	13 (13.5)	11 (15.5)	2 (2.0)	
Combined, n (%)	8 (8.3)	7 (9.9)	1 (4.0)	
Unknown, n (%)	4 (4.2)	2 (2.8)	2 (8.0)	
**Medical history**				
Hypertension, n (%)	35 (36.5)	27 (38.0)	8 (32.0)	0.590
History of smoking, n (%)	37 (38.5)	29 (40.8)	8 (32.0)	0.435
Diabetes, n (%)	15 (15.6)	11 (15.5)	4 (16.0)	0.973
Acute myocardial infarction, n (%)	16 (16.7)	13 (18.3)	3 (12.0)	0.467
COPD, n (%)	10 (10.4)	8 (11.3)	3 (8.0)	0.646
Coronary artery disease, n (%)	18 (18.8)	15 (21.1)	3 (12.0)	0.315
**Laboratory values at admission**				
Catalase, median (IQR)	8.25 (7.64-8.81)	7.98 (7.61-8.75)	8.69 (7.96-9.86)	**0.006**
pO2 mmHg, median (IQR)	61.15 (25.68-215.00)	64.00 (27.00-243.00)	38.00 (20.50-189.00)	0.228
pCO2 mmHg, median (IQR)	15.70 (7.48-23.85)	15.60 (7.40-21.15)	20.00 (10.50-28.60)	0.058
pH, median (IQR)	7.18 (7.08-7.23)	7.19 (7.12-7.25)	7.08 (6.97-7.18)	**0.002**
Sodium mmol/l, median (IQR)	138 (136-140)	138 (136-140)	138 (135-140)	0.301
Potassium mmol/l, median (IQR)	3.85 (3.48- 4.54)	3.76 (3.48-4.33)	4.50 (3.66-5.03)	0.077
Bicarbonate mmol/l, median (IQR)	8.30 (5.88-10.93)	7.90 (5.10-9.85)	11.40 (6.33-11.10)	**0.002**
Base excess mmol/l, median (IQR)	9.10 (6.00-12.75)	8.20 (5.80-11.90)	12.30 (9.10-16.55)	**0.003**
Lactate mmol/l, median (IQR)	7.20 (4.90-9.90)	6.90 (4.65-8.75)	10.05 (7.40-11.73)	**0.002**
Creatinine, mg/dl, median (IQR)	1.27 (1.04-1.54)	1.25 (1.01-1.52)	1.31 (1.05-2.08)	0.182

Blood Urea Nitrate, mg/dl, median (IQR)	15.10 (12.00-19.90)	14.80 (11.20-19.90)	12.75 (12.38-20.60)	0.408
NT-proBNP, pg/ml, median (IQR)	441 (118-1862)	334 (93-1437)	1296 (239-2418)	**0.028**
S100B, µg/l, median (IQR)	0.10 (0.06-0.21)	0.09 (0.06-0.12)	0.38 (0.17-1.48)	**< 0.001**
Neuron specific enolase, µg/l, median (IQR)	30.65 (17.85-74.18)	28.30 (14.80-46.30)	87.90 (39.65-189.50)	**< 0.001**

Abbreviations: COPD: chronic obstructive pulmonary disease, pO2: partial pressure of oxygen, pCO2: partial pressure of carbon dioxide, IQR: interquartile range, n: number, yr: years
